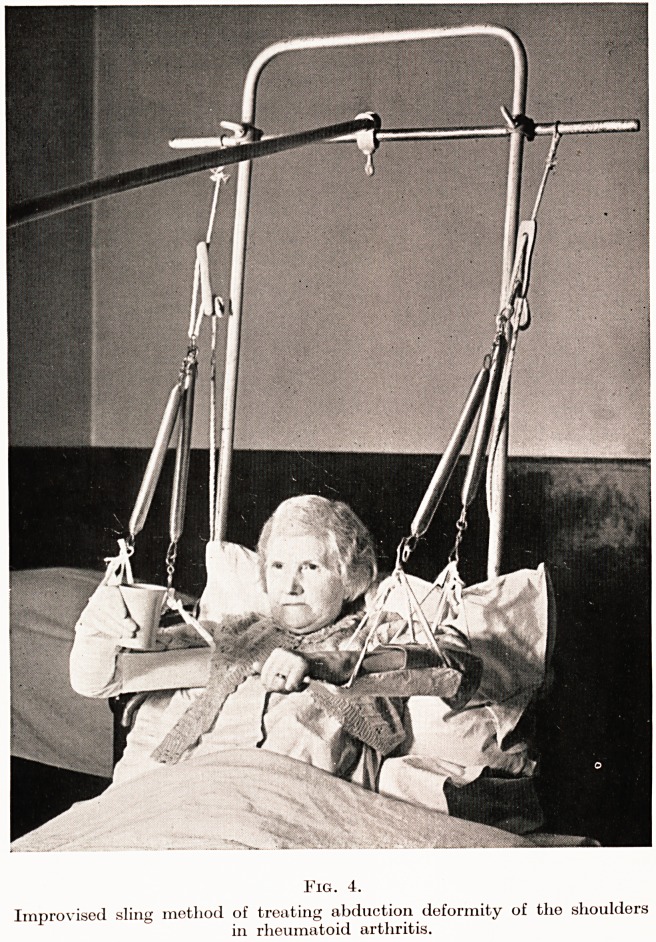# The Diagnosis and Treatment of Rheumatoid Arthritis

**Published:** 1946

**Authors:** G. D. Kersley

**Affiliations:** Assistant Physician, Royal United Hospital, Bath; Physician, Royal National Hospital for Rheumatic Diseases, Bath


### THE DIAGNOSIS AND TREATMENT OF RHEUMATOID ARTHRITIS

BY

Dr. G. D. Kersley, M.D., F.R.C.P.

*Assistant Physician, Royal United Hospital, Bath;*
*Physician, Royal National Hospital for Rheumatic Diseases, Bath.*

An Address to the Bristol Medico-Chirurgical Society on 13th March, 1946.

In a large percentage of cases, a diagnosis of Rheumatoid Arthritis can be made from the far end of the room ; but in some 5 per cent. it may be so difficult that three equally eminent physicians might make three different diagnoses, entailing quite different treatment for the same case. The remaining 95 per cent., in which most would agree to the diagnosis, may be sub-divided into two syndromes, typically distinct but with intermediate forms. Their recognition is important, as both prognosis and treatment differ.

Idiopathic rheumatoid arthritis occurs in the female sex in 70 per cent. of cases and usually in the asthenic type. The onset often follows shock, worry or fatigue. It commences insidiously ; wasting of muscles and trophic changes in the skin are very marked and are sometimes the first sign of the condition ; the small peripheral joints are first and usually symmetrically involved. There is an early loss in weight : there is usually a microcytic anaemia. The sedimentation rate is always raised and does not undergo rapid fluctuations ; the Arneth count of the lobes of the polymorphonuclear cells is often normal ; X-rays show a generalized osteoporosis.

The non-specific infective or secondary type of rheumatoid (focal arthritis) occurs equally frequently in either sex and there is no characteristic diathesis. It often occurs within a month, typically in ten to fourteen days, after an infection—sore throat, quinsy, antrum infection, appendix, etc. : or on examination an obvious septic focus such as an apical abscess at the root of a tooth is discoverable. Its onset may be more acute, starting first in one of the larger joints and then spreading to the smaller peripheral ones, but usually less symetrically than in the "idiopathic" type. Only those muscles are wasted which act on the joints involved, and trophic changes are less marked. There may be little or no loss of weight. A microcytic anaemia is usually present ; the sedimentation rate is very rapid but may fluctuate considerably within a few weeks ; the Arneth count usually shows a shift to the left denoting more immature white cells in the circulation ; osteoporosis is mainly confined to those bones in the region of the involved joints. In this syndrome, with eradication of sepsis and sometimes with gold-therapy, the prognosis is often excellent, whereas in the idiopathic type the removal of septic foci usually has little effect unless they be gross enough to jeopardize the general health of the patient.

Mrs. H., age thirty-four, a somewhat introspective, highly strung woman, had suffered from a quinsy in February, 1944 and again in September. In November, 1944, she had another sore throat, while at the same time she was working very hard as a school-teacher and was much concerned about the "fly-bombs". In December there was pain and swelling in the right shoulder, followed rapidly by involvement of the knees, ankles, wrists and fingers. There was little loss in weight. In April, 1945, the tonsils were removed, and this was followed by a course of myocrisin totalling 1.9 gm. At this time the sedimentation rate was 80 mm. W. By November, 1945, the sedimentation rate had fallen to 7.5 mm. W. and she was quite fit except for slight stiffness and occasional twinges of pain in the wrists.

*Differential Diagnosis.*—There are at least five fairly common conditions, which, though typically quite distinct, may at times simulate the secondary type of rheumatoid arthritis. They are gout, polyarticular osteoarthritis, peri-articular fibrositis, ankylosing spondylitis, and specific infective arthritis.

Typical Gout is easily diagnosed with its family history of the condition in 50 per cent., the sex incidence, the past history of acute attacks followed by complete remissions, and the sthenic build. There is no loss of weight. The blood uric acid is raised in 70 per cent of cases; the sedimentation rate is frequently raised but rapidly varying; anaemia is absent; X-rays of the hands or feet show punched-out areas (the hands should always be X-rayed with a control, even if there is no obvious involvement in this area). Full doses of colchicum provide a therapeutic test: the dose should be sufficient to cause slight diarrhoea before the absence of effect can be considered in establishing a diagnosis. But gout does occur in women and is then particularly liable to be atypical and without acute attacks. Also punched-out areas are seen in X-rays of rheumatoid arthritics, though in these cases the area is usually co-incident with erosion of the over-lying cartilage. (Figs. 1 and 2.)

In Polyarticular Osteoarthritis the joints of the hands are typically nodular instead of fusiform. The skin is often red, instead of blanched and sweating; there is no loss in weight. The sedimentation rate is not as a rule raised and X-rays may show the typical Heberden's nodes arising from the periosteum in the distal phalanges.

Peri-articular Fibrositis frequently occurs around the finger joints of adults who have in the past suffered from rheumatic fever. This raises again the question of the inter-relationship of the rheumatic diseases, a question re-opened recently by the American work on lesions found post-mortem in the hearts of rheumatoid patients. These cases of fibrositis do not, however, as a rule, progress to a

PLATE I.

[*By courtesy of Dr. G. D. Steven*

FIG. 1.

Erosions in gout : note fine cortical layer largely intact.

[*By courtesy of Dr. G. D. Steven*

FIG. 2.

Erosions in rheumatoid arthritis : note surface erosion.

### PLATE II.

A

B

C

Fig. 3.

Treatment of flexion deformity of the knees by means of serial plasters (A, B, and C.).

rheumatoid syndrome, and the sedimentation rates and X-rays are usually normal. Their treatment is extremely unsatisfactory.

Spondylitis Ankylopoietica, occurring in men three times more frequently than in women, and involving mainly the spine and sacro-iliac joints, often commences with symptoms of sciatica. It does sometimes, however, start with pain and swelling in the peripheral joints, and then the loss of weight, raised sedimentation rate and osteoporosis in the X-rays make it for a time indistinguishable from rheumatoid arthritis. In America this type seems to be more common than in this country, and Americans therefore frequently refer to spondylitis ankylopoietica as rheumatoid arthritis of the spine. Ankylosing spondylitis usually reacts poorly to gold-therapy but is frequently helped by X-ray treatment.

Specific Infective Arthritis, of which Gonorrhoeal Arthritis is one of the best examples, is often indistinguishable from the secondary type of rheumatoid arthritis except by isolation of the organism concerned. However, it reacts well to penicillin and frequently also to sulphonamides and hyperthermy, all of which are useless in rheumatoid arthritis. Attacks of iritis, conjunctivitis, fasceitis and teno-synovitis are suggestive of a specific rather than a non-specific origin.

*Ætiology.* The most widely held view of our conception of the ætiology of rheumatoid arthritis may be summed up in the words of Professor Stanley Davidson[1] as "an abnormal immunological reaction of the host to an infection," and, one might add, perhaps to certain other traumata : physical, dietetic and above all psychological. The factors proven statistically to be significant in connection with the onset of rheumatoid arthritis are infection (probably not one particular infection), fatigue and exposure, worry and shock.

*Treatment.* This can be considered under the headings of general treatment, elimination of sepsis, physical and orthopaedic treatment, and the use of drugs.

In every active case the patient should at first be put to bed and given as complete physical and mental rest as possible ; he should receive a good nourishing diet with plenty of vitamins and iron and should live under the best hygienic conditions.

In "idiopathic" rheumatoid arthritis only gross sepsis should be eliminated : but in the "focal" type a careful search should be made both by history-taking and examination for sepsis in the teeth, sinuses, tonsils, gall-bladder, appendix, and prostate or cervix. All teeth should be X-rayed, and any that are dead, crowned or unerupted should be looked at particularly critically. No tooth should, however, be removed unless there is definite evidence of infection, and not more than one should be extracted at a time, at all events in the first instance. Where, after careful removal and fixation of the crown in wax, a culture of the same organism can be obtained from a number of points on the root, and where the patient shows a reaction to the extraction, an autogenous vaccine should be prepared in high dilution, e.g., 100 million organisms to the c.cm. If there is clinical progress for a time, followed by a phase in which the condition becomes stationary, the vaccine should be used. The routine use of vaccine is to be deprecated.

Under the heading of physical and orthopaedic treatment should be considered: (*a*) the balancing of rest and exercise, including the prevention of deformity; (*b*) re-education of the capillary circulation; (*c*) counter-irritation; (*d*) X-ray therapy; and (*e*) correction of deformity already present. As a general rule, during the acute and sub-acute stages the affected joints should be rested on light splints. These are removed daily for movement once through their full range, for the application of heat, possibly some gentle massage of the effleurage type for the whole limb, and static contraction and perhaps faradic stimulation for the muscles acting on the joint. The movements are best carried out in a warm pool, or failing this in a warm room, in Guthrie-Smith suspension slings.

For re-education of the circulation, contrast heat and cold is employed followed by a good rub-down, the aim being to produce a feeling of "glow" and well-being. The temperatures required to produce this will vary with each patient and, if successful, will increase in range as the treatment progresses. Counter-irritation may be applied in many ways ranging from a simple Scott's dressing or kaolin poultice containing cantharides to the cautery or an erythema dose of ultra-violet light.

The correction of flexion deformity in the knees is best carried out by the application of serial plasters, each being applied with the maximum amount of extension possible and being divided after twenty-four hours so that it can be removed daily for treatment (Fig. 3). Usually after about ten days there has been a gain in extension of some ten degrees and a new plaster is then put on to take up the slack. Correction of deformity in the hands is more difficult, but rest-plasters combined with exercises and occupational therapy will often help, though occasionally finger-traction is indicated. For the shoulders, treatment in sling-suspension with springs is by far the most satisfactory method of increasing abduction and rotation. (Fig. 4.)

Analgesics in full doses are of value both for promoting sleep and improving the appetite by the relief from pain, and it is difficult to beat the time-tried mixture of aspirin or calcium aspirin: aspirin gr. x, phenacetin gr. v, and caffeine gr. i, given three or four times a day if necessary. The sulphonamides and penicillin are useless in true rheumatoid arthritis and any effect from these indicates specific infection. Likewise, hyperthermy is only of value in gonococcal cases. Gold is by far the most valuable form of drug-therapy, but should only be used after a preliminary period of rest and treatment on the general lines indicated above and when the presence of activity

PLATE III.

Fig. 4.

Improvised sling method of treating abduction deformity of the shoulders in rheumatoid arthritis.

is indicated by a raised sedimentation rate. Progressive or severe renal or liver damage and the presence of eczema are contra-indications to its use, and during its administration X-ray treatment and ultra-violet light should be employed with caution, if at all, owing to the sensitizing effect on the skin. Sodium aurothiomalate (myocrisin), and more recently the calcium salt (auro-calcium), are the preparations most used, and the dosage employed is much smaller than was the case some years ago. One c.cm of collosol calcium given by injection at the time seems to reduce the incidence of toxic reactions. The onset of dermatitis, albuminuria or blood in the urine, stomatitis, severe joint reactions with fever, colitis or severe leucopenia (a fall in the white cell count below 4,000, affecting mainly the granulocytes), are indications for stopping the drug. Provided that such mishaps do not occur, an average course of auro-calcium could be planned on the following lines : 0.01 gram, 3 doses ; 0.025 gram, 3 doses ; 0.05 gram, 18 doses—total 1.0 gram.

From the above outline of the treatment of the " rheumatoid syndromes " two facts stand out clearly : that where possible, rheumatoid cases should be sub-divided into " idiopathic " and " focal " types in order to assess the likely significance of infection ; and that, in both types, in the present position of our knowledge, a wide view should be taken of treatment and every aspect receive due consideration in drawing up a plan of campaign.

REFERENCE.

[1] *Glasgow Med. Jour.*, 1943, p. 140.

## Figures and Tables

**Fig. 1. f1:**
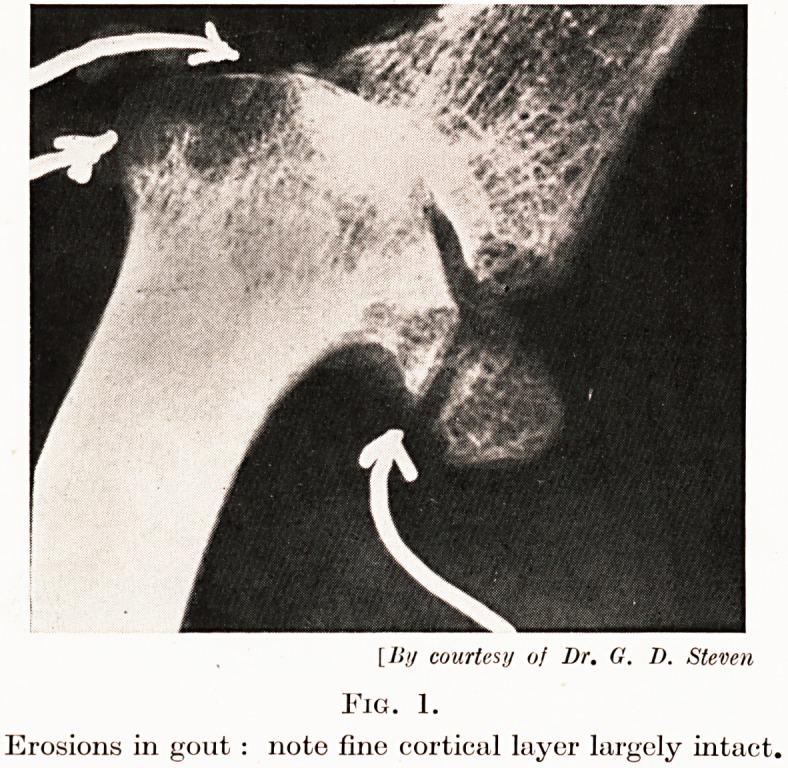


**Fig. 2. f2:**
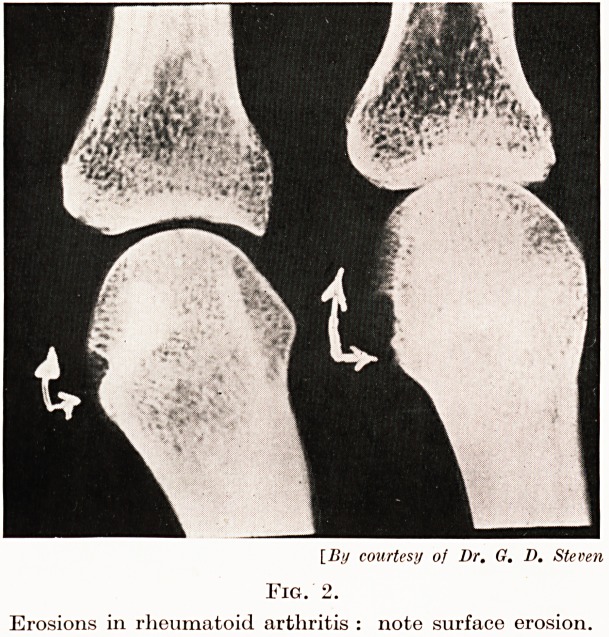


**Fig. 3. f3:**
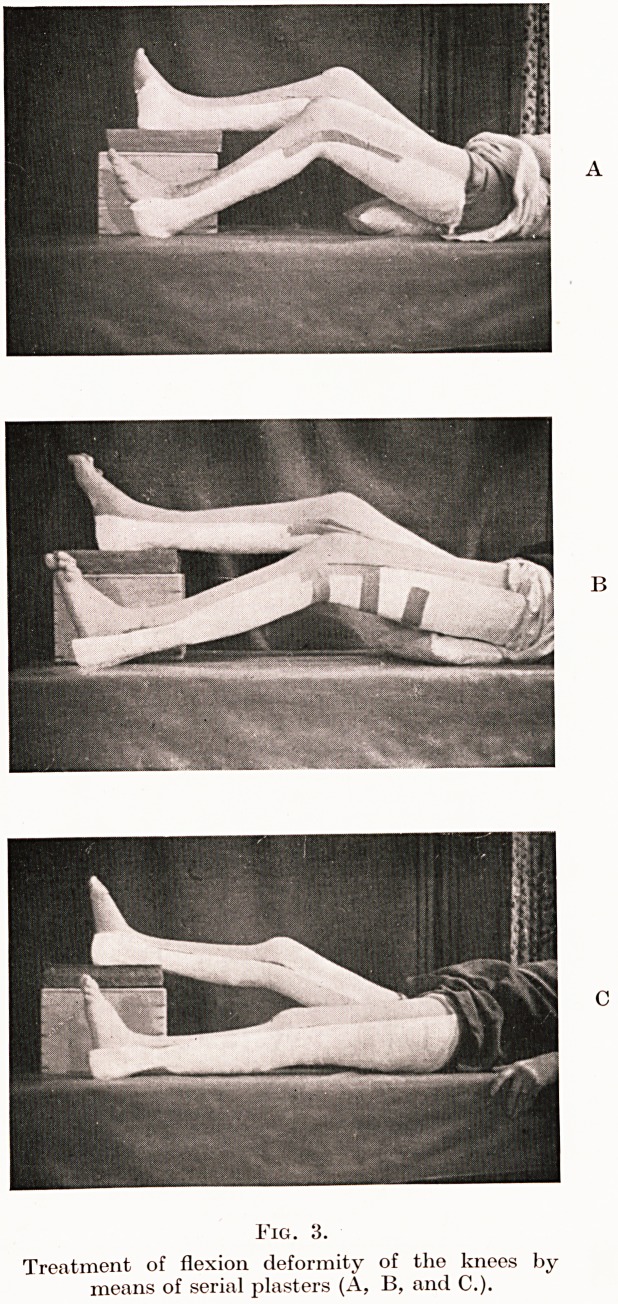


**Fig. 4. f4:**